# Transcriptome Analysis of a Petal Anthocyanin Polymorphism in the Arctic Mustard, *Parrya nudicaulis*

**DOI:** 10.1371/journal.pone.0101338

**Published:** 2014-07-17

**Authors:** Timothy Butler, Cynthia Dick, Matthew L. Carlson, Justen B. Whittall

**Affiliations:** 1 Department of Biology, Santa Clara University, Santa Clara, California, United States of America; 2 Biological Sciences Department, University of Alaska Anchorage, Anchorage, Alaska, United States of America; Montreal Botanical Garden, Canada

## Abstract

Angiosperms are renown for their diversity of flower colors. Often considered adaptations to pollinators, the most common underlying pigments, anthocyanins, are also involved in plants’ stress response. Although the anthocyanin biosynthetic pathway is well characterized across many angiosperms and is composed of a few candidate genes, the consequences of blocking this pathway and producing white flowers has not been investigated at the transcriptome scale. We take a transcriptome-wide approach to compare expression differences between purple and white petal buds in the arctic mustard, *Parrya nudicaulis*, to determine which genes’ expression are consistently correlated with flower color. Using mRNA-Seq and *de novo* transcriptome assembly, we assembled an average of 722 bp per gene (49.81% coding sequence based on the *A. thaliana* homolog) for 12,795 genes from the petal buds of a pair of purple and white samples. Our results correlate strongly with qRT-PCR analysis of nine candidate genes in the anthocyanin biosynthetic pathway where chalcone synthase has the greatest difference in expression between color morphs (P/W = ∼7×). Among the most consistently differentially expressed genes between purple and white samples, we found 3× more genes with higher expression in white petals than in purple petals. These include four unknown genes, two drought-response genes (CDSP32, ERD5), a cold-response gene (GR-RBP2), and a pathogen defense gene (DND1). Gene ontology analysis of the top 2% of genes with greater expression in white relative to purple petals revealed enrichment in genes associated with stress responses including cold, drought and pathogen defense. Unlike the uniform downregulation of chalcone synthase that may be directly involved in the loss of petal anthocyanins, the variable expression of several genes with greater expression in white petals suggest that the physiological and ecological consequences of having white petals may be microenvironment-dependent.

## Introduction

The loss of floral anthocyanins in white flowers provides an unparalleled opportunity to examine the genes underlying a distinctive phenotypic transition. The diversity of flower colors among angiosperms is most often attributed to the preferences of their pollinators [1], [2], [3], [4], [5], [6], yet the underlying anthocyanin pigments are also involved in a diversity of stress-related functions not directly related to pollinator attraction (e.g., UV-protection, drought tolerance, cold stress and herbivore resistance; [7], [8], [9]). To disentangle the roles of pollinator and non-pollinator agents of selection on flower color, we studied a habitat in interior Alaska, where pollinators are exceedingly rare [10], [11] and non-pollinator agents of selection are predicted to be paramount [12].

**Figure 1:**
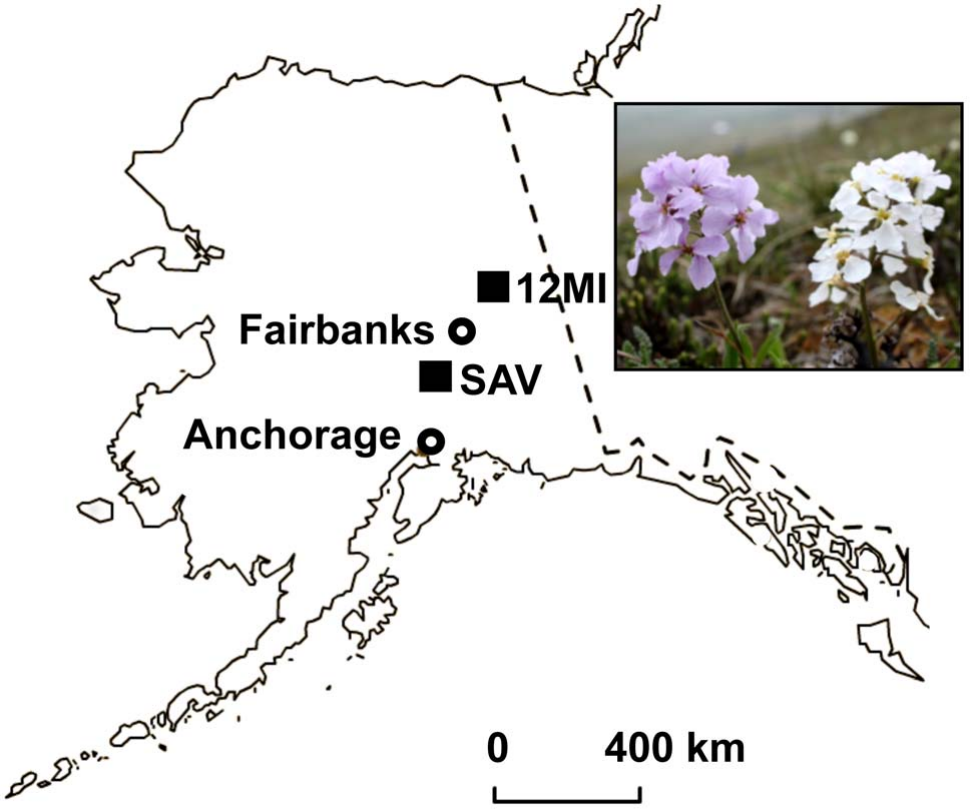
Arctic mustard flower color polymorphism and sampling localities. The arctic mustard, *Parrya nudicaulis,* exhibits variation in petal anthocyanins within populations across Alaska. RNA was collected from pooled buds of purple- and white-flowered individuals at Savage River (SAV) and 12 Mile Summit (12 MI) indicated with filled squares. Landmark cities are indicated with open circles.

The purple-white flower color polymorphism in the arctic mustard, *Parrya nudicaulis*, offers a unique perspective on potential non-pollinator agents of selection (Fig. 1; [13]). Pollinator observations in interior Alaska and along the Arctic Coastal Plain confirm the relative rarity of pollinators [14], making it unlikely that pollinator preferences alone are responsible for such geographically widespread variation. Since the frequency of white individuals is positively correlated with the length of the growing season, we suspect climatic factors (or correlated, non-pollinator selective agents) interacting with the petal color contribute to the maintenance of this polymorphism [13]. We have also detected selection against individuals with lighter colored petals along the Arctic Coastal Plain, but not in interior Alaska where the climate during the growing season is more benign [14].

Previously, a candidate gene approach focusing on nine genes in the anthocyanin biosynthetic pathway revealed a 24-fold expression reduction of chalcone synthase (CHS) in white petals, with no comparable expression change in the sepals or leaves [13]. However, our candidate gene approach was limited to the anthocyanin biosynthetic pathway genes and was not capable of revealing any consequences of the loss of floral anthocyanins outside of the genes immediately involved in this biochemical pathway. Although genomic resources for *P. nudicaulis* are limited (prior to this study, Genbank contained only 14 accessions), the close relationship to *Arabidopsis thaliana* (approximately 89% nucleotide similarity in coding regions) allows us to utilize a wealth of genome-scale information such as TAIR’s RefSeq database [15], the Gene Ontology database [16], the ATTED-II gene co-expression database [17], a collection of functional gene networks in ARANET [18], and the Arabidopsis Co-expression Tool [ACT [19]].

Transcriptome analysis can generate the sequences of most genes present in a target tissue at a particular developmental stage and simultaneously estimate their expression levels. Expressed sequence tags (ESTs) and serial analysis of gene expression (SAGE) have been used as a form of transcriptome analysis for decades [20], [21], [22], [23], but the amount of Sanger sequencing necessary for accurate expression estimates from ESTs and even SAGE was prohibitively expensive for all but a few model organisms. The advent of massively parallel sequencing technologies has made transcriptome analysis possible for many non-model species [24], [25], [26], [27]. Dubbed mRNA-Seq on the Illumina platform (Illumina, San Diego, CA), the production of millions of relatively short reads (40–300 bp long) is technically feasible and relatively inexpensive for any organism in which RNA can be adequately preserved. Acquiring the data is relatively straightforward [26] compared to the challenge of re-assembling the reads into contiguous sequences, annotating the contigs, and accurately estimating expression. Although such studies have been largely restricted to model organisms with complete reference genomes such as human, mouse, yeast, *A. thaliana*, and rice [28], [29], [30], [31], [32], transcriptome assembly and expression analysis without a reference genome (*de novo*) has been adopted in many non-model organisms [24], [27], [33]. In eukaryotes, one of the many hurdles in *de novo* transcriptome assembly is differentiating orthologous and paralogous sequences, a complication that can be exacerbated by recent whole-genome duplication events (i.e. polyploidy).

We first investigate the ploidy levels of *P. nudicaulis* in our focal populations. We then describe the *de novo* assembly and expression analysis of the petal bud transcriptome of *P. nudicaulis* from purple- and white-flowered individuals from two populations (hereafter referred to simply as “transcriptome”). We validate our assembly and expression results for purple and white petals using quantitative real-time PCR (qRT-PCR) for nine candidate genes in the anthocyanin biosynthetic pathway. Broadening our perspective to the transcriptome has uncovered several unexpected, yet consistently differentially expressed genes between purple and white petals potentially involved in plants’ stress response. Some of these genes may reflect the consequences of the loss of floral anthocyanins and could provide clues to additional targets of selection by non-pollinator agents. Our transcriptome approach has produced several testable hypotheses regarding the nature of the genes with differential expression in the petal bud that are consistently correlated with flower color variation in *P. nudicaulis*.

## Materials and Methods

### Genome Size Estimation

To estimate genome size, we used flow cytometry on fresh leaves from one to three individuals representing three populations of *P. nudicaulis* including the two populations used in subsequent transcriptome analysis (Table 1). Tissue was kept on ice until it could be homogenized. Cells were lysed in Galbraith’s buffer [34] and nuclei were stained with propidium iodide (50 µg/ml) and RNase (50 µg/ml). Flow cytometry measurements were performed on a Becton Dickinson FACScan flow cytometer equipped with a 488 nm argon laser. Genome size was estimated in comparison to *A. thaliana* [2C = 0.32 pg; [35]]. Peak positions and genome size estimates were similar whether *A. thaliana* was used as an internal or external standard. Conversion from picograms of diploid nuclear DNA to base pairs followed Dolezel et al. [36]: 1 pg diploid nuclear DNA = 978 million base pairs.

**Table 1:** Sample localities and genome size estimates for *P. nudicaulis.*

Latitude	Longitude	Population	Individual	Genome Size –2C (pg)
68.47°N	–149.56°W	Galbraith	1	2.08
			2	2.03
			3	1.88
65.40°N	–145.99°W	12 Mile	1	2.09
			2	2.17
65.47°N	–145.43°W	Eagle Peak	1	2.04
			Mean±1SE	2.08±0.039

SE = Standard Error.

### RNA Sampling

Petal buds were collected at two Alaskan *P. nudicaulis* populations: Savage River in Denali National Park and the 12 Mile Summit located approximately 200 km northeast of Fairbanks (Fig. 1). Denali National Park granted permits for sampling at the Savage River population (Denali Permit number DENA-2008-SCI-0024). At each population, petals from three to five individuals per color morph were pooled in the field and immediately frozen in liquid nitrogen until total RNA could be extracted using the RNeasy Plant Mini Kit (Qiagen, Valencia, CA). Only the Savage River samples were used for *de novo* petal bud transcriptome assembly. Samples from both Savage River and 12 Mile Summit were used for expression analysis (see below). RNA integrity numbers were determined on a Bioanalyzer (Agilent Technologies, Santa Clara, CA) and ranged from 5.7–9.7 (mean = 7.85). The quality was sufficient to proceed with the mRNA-Seq library preparation [37].

### Library Preparation and Illumina Sequencing

Total RNA extracts were prepared for mRNA-Seq on the Illumina Genome Analyzer II following the manufacturer’s suggested protocols (Illumina). Briefly, poly-A+ RNA was isolated from tRNA and rRNA using poly-T oligonucleotides attached to magnetic beads. The mRNA was then eluted and fragmented using a proprietary fragmentation buffer relying on divalent cations and high temperature. cDNA was synthesized from the mRNA templates using random hexamers and adapters were ligated to each blunt end cDNA. Fragments of 200 bp (+/−25 bp) were gel extracted from an agarose gel and PCR amplified for 15 cycles. Each of the four mRNA-Seq libraries were loaded on individual lanes of the Illumina GAII (UC Davis Genome Center), followed by 40 cycles of single-end sequencing-by-synthesis reactions. Reads are available from Genbank’s Sequence Read Archive (Accession# SRA028419).

### *De Novo* Assembly

**Figure 2:**
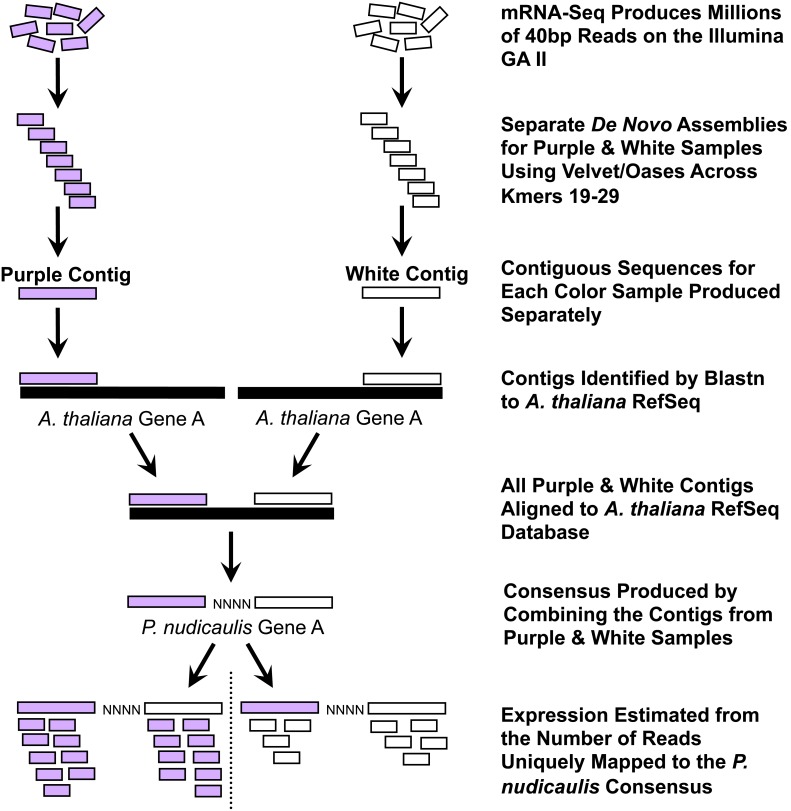
*De novo* transcriptome analysis pipeline. A seven-step bioinformatics pipeline for *de novo* transcriptome analysis of mRNA-Seq data. For each color sample, multiple assemblies using a range of kmer values were conducted separately using Velvet/Oases (not shown). The kmer parameter providing the greatest average contig length per gene for both purple and white was used to create the consensus sequence that became our reference for estimating expression. Expression was measured as reads per kilobase exon per million uniquely mapped reads (RPKM). In the example illustrated, the purple sample has twice the expression of the white sample assuming an equal number of uniquely mapped reads across the entire transcriptome.

Although *P. nudicaulis* shares ∼89% DNA sequence similarity to *A. thaliana* in several coding regions [13], it is too divergent to accurately perform a reference-guided assembly (Butler and Whittall, unpublished data). Therefore, we developed a bioinformatics pipeline for *de novo* transcriptome assembly and expression analysis (Fig. 2). We used the *de novo* assembler Velvet v.1.0.03 and Oases v.0.1.11. The latter is specifically designed for the uneven coverage depths and alternative splicing common in transcriptome analysis and can generate multiple isoforms per contig to account for different alleles, paralogs, and splice variants [38]. Using the unfiltered fastq files produced by the Illumina pipeline, we compared assemblies across a range of Velvet/Oases parameter values (kmers 19–29) for the two Savage River samples. The upper kmer limit was based on recommendations in Zerbino [maximum kmer = (read length–10); [39]]. The lower kmer limit was constrained by computational time.

Contigs were then identified using nucleotide blast to the *A. thaliana* RefSeq database [TAIR version 9; [15], [40]] limiting our results to those contigs with E-values <10^−10^. Reciprocal best-hit nucleotide blast with the contigs as the database and the *A. thaliana* RefSeq nucleotides as the query was used to confirm orthology using the same E-value cutoff of less than 10^−10^ [41]. Contigs with E-values >10^−10^ and those that did not pass the reciprocal best-hit blast criteria were removed. Multiple contigs per gene for both purple and white samples were aligned to the *A. thaliana* Reference Sequence for each individual kmer run using muscle v.3.8.31 [42]. We then removed the *A. thaliana* sequences and produced a consensus sequence representing all variable sites among the *P. nudicaulis* contigs as ambiguities. Any missing sequence regions in the *P. nudicaulis* consensus were padded with n’s (Fig. 2). For each gene, we chose the longest Velvet/Oases kmer assembly that was unlikely to represent misassembly or misalignment based on the proportion of ambiguities (excluding n’s) in the consensus as our reference for expression analysis. An earlier attempt to use all the contigs from all kmer analyses produced excessive ambiguities due to occasional misassembly or misalignment (Butler and Whittall, unpublished data). This approach has also been criticized because of the likelihood of assembling chimeric consensus sequences (D. Zerbino, pers. comm.).

As part of our quality control, we examined 566 alignments that produced consensus sequences with more than 1.9% ambiguities - the maximum expected intraspecific nucleotide variation in coding sequence based on previous sequence comparisons on a much larger sampling of individuals across the species’ range [13]. Among these 566 alignments, the most common source of variation was misalignment of the contigs, which was corrected by hand or if not possible, the culprit contig was removed from the alignment altogether. Occasionally, the elevated ambiguity was caused by alignments with nearly identical coding regions, but highly divergent UTR sequences. These were assumed to be paralogs and since their coding regions were highly conserved and they passed our reciprocal best-hit blast filter, their similar function and likely duplication since the split with *A. thaliana* justified their inclusion in the consensus sequence. Another frequent problem in the subset of alignments with more than 1.9% ambiguity was contigs with highly divergent coding sequences. Although these genes passed our reciprocal best-hit nucleotide blast criteria, one set of contigs was always much more similar to the *A. thaliana* coding sequence, so the more divergent contigs were removed. Consensus sequences created from these individually examined alignments were included along with the rest of the reference sequences with less than 1.9% ambiguity for expression analysis.

### Expression Analysis

Expression levels were estimated by mapping reads from both Savage River and 12 Mile Summit populations back onto the *de novo* assembled reference transcriptome using the Mosaik assembler since it utilizes IUPAC ambiguity codes in the reference sequences and allows for a range of mismatches per hash (Michael Stromberg, Boston University). Mosaik parameters followed the recommendation of the authors - a hash length of 14 and two allowed mismatches. Qualitatively similar results were obtained with a hash length of 17 and four mismatches (Whittall, unpublished data). Only uniquely mapped reads were used in comparing expression as reads per kbp exon per million reads mapped (RPKM). Genes in which any of the four samples had very low expression (less than 10 reads mapped) were removed since the low number of reads produced spurious estimates in the purple-white fold-change calculation.

We used linear regression (Jmp 4.0, SAS Institute, Cary, North Carolina) to compare our mRNA-Seq expression results to a pre-existing qRT-PCR dataset using the same four RNA samples for nine anthocyanin biosynthetic pathway-related genes [13]. The RPKM expression estimates and the linearized qRT-PCR data [(1+Efficiency)^ΔCT^] were both log_10_-transformed to meet assumptions of normality. We also compared the mRNA-Seq results to the qRT-PCR estimates for P/W fold-change using linear regression after removing the CHS outliers.

In order to identify *consistently* differentially expressed genes, we compared the paired purple and white samples from Savage River to those from 12 Mile Summit. To visualize genes with purple expression greater than white (P>W) and genes with white expression greater than purple (W>P) on a single continuous axis, we calculated the relative difference (RD) in expression between purple and white samples using RPKM values for P and W samples as RD = (P – W)/[max(P,W)]. Genes in which the expression difference between purple and white was greater than 50% of the larger of the two (RD>0.5 or RD<–0.5) were considered differentially expressed. We then determined the consistency between the relative difference at each population as: Consistency = RD_SavageRiver_ – RD_12MileSummit_. We applied a threshold for consistency of ±0.25. If samples were evenly distributed across the consistency spectrum (–1.00 to 1.00), this cutoff represents the most consistent quartile of all genes. Using these metrics, we identified two sets of genes that are consistently differentially expressed (P>W and W>P).

### qRT-PCR Validation of Consistently Differentially Expressed Genes

We examined seven consistently differentially expressed genes using qRT-PCR on a larger set of RNA samples. Among genes with P>W, we chose the most differentially expressed gene after chalcone synthase, GDSL-motif lipase/hydrolase family protein (AT5G45950; P/W = 2.80). For W>P, we chose six genes based on their W/P value, overall expression levels and functional annotation in *A. thaliana* homologs. The W>P set consists of a 7SL RNA gene with the greatest expression difference (AT2G31141; W/P = 12.29), one gene with unknown function in *A. thaliana* (AT1G10020), three genes of ecological interest [drought response (CDSP32; AT1G76080), dehydration response (ERD5; AT3G30775), and defense against pathogens (DND1; AT5G15410)], and a consistently differentially expressed gene with the highest overall expression (EXL5; AT2G17230). For each gene, we examined expression in the same four samples used for transcriptome analysis and added another eight similarly pooled samples representing a range of floral developmental stages (two purple and six white from bud to anthesis stages). All additional samples are from the same two populations used in the transcriptome analysis. TaqMan qRT-PCR assays were used to validate the transcriptome expression levels following the ΔΔC_T_ method [43]. Fragment lengths averaged 72.25 bp (range 70–75 bp). Amplification efficiencies for each locus were determined from a standard curve [mean = 0.963 (range 0.926–1.000)] and expression was standardized in comparison to the endogenous control (GAPC2; AT1G13440) as described in Dick et al. [13]. We then compared standardized expression between purple and white samples by relativizing to a single purple sample (Savage River 23).

### Gene Ontology Analysis

We tested for enrichment in gene ontology terms among the top 2% of differentially expressed genes within our consistency threshold (N = 92) for P>W and W>P. Using a 2% cut-off produced a set of genes with a large enough sample size to detect enrichment since approximately 1/3 of the most differentially expressed genes were unannotated in *A. thaliana*. We tested specifically for enrichment in gene ontology (GO) terms associated with Biological Process using the hypergeometric test with a Benjamini and Hochberg correction for multiple testing (FDR = 0.01) using the GO Stats Enrichment/Depletion Statistical Assessment in the GO Toolbox [[16] GO Toolbox website. Available: http://genome.crg.es/GOToolBox/. Accessed 2014 June 22]. We compared the top 2% of consistently differentially expressed genes with P>W and W>P to a null set of the remaining 10,802 genes with at least 10 reads in the expression analysis (RPKM>∼1).

## Results

### Genome Size Estimation

Since recent polyploidy can complicate transcriptome analysis due to genome-wide paralogy, we examined the genome size of six *P. nudicaulis* samples from three populations using fresh leaf tissue extracts on a flow cytometer. The size of the diploid *P. nudicaulis* nuclear genome was consistently estimated at 2.08 pg (Table 1). Our genome size estimates suggest the haploid genome measures approximately 1.02 Gbp [44]. There was no evidence of within or between population polyploidy, which reduces complications from paralogy during the transcriptome analysis.

### Illumina Sequence Results and *De Novo* Transcriptome Assembly

Four lanes of 40 bp single-end reads from the Illumina GAII generated between 15.3–28.7 million reads (mean = 21.6 million reads) totaling 3.5 Gbp (Table S1 in File S1). Since the assembly parameters can dramatically affect the length and reliability of contigs, we ran Velvet/Oases *de novo* assembly on each of the two Savage River samples separately across kmers 19–29. This analysis produced a nearly two-fold range in the number of contigs produced for each sample (purple 27,351–47,884; white 22,772–40,228 contigs). Oases isolated 1–35 isoforms of each contig (mean = 1.4 isoforms per gene). Blastn to the *A. thaliana* RefSeq database identified 11,483 genes from the purple sample and 10,586 genes for the white sample. To improve our confidence in the transcript identification, we conducted reciprocal best-hit nucleotide blast filtering on both the purple and white assemblies. We then merged them to create a transcriptome of 12,795 unique genes. Approximately 72.5% of these genes had more than one contig per gene (mean = 1.475 contigs per gene; range 1–11 contigs per gene). After combining contigs from purple and white samples into a single alignment for each kmer assembly, we chose the kmer that produced the longest assembly (per gene) that was unlikely to contain misassembly or misalignment errors (less than 1.9% ambiguities in the consensus). The majority of contigs in the final transcriptome came from kmers 19 and 21 (∼58%). On average, we sequenced 722 bp of each gene, assembled 49.81% of each gene’s coding sequence, and sequenced the entire coding sequence for over 2,000 genes (∼16% of the assembled transcriptome).

For the nine anthocyanin biosynthetic pathway candidate genes, we sequenced an average of 89.0% of their coding sequences plus an average of 103 bp of their UTRs (Table 2). For two anthocyanin biosynthetic pathway genes, CHS and flavonol synthase, we obtained the entire coding sequence and greater than 100 bp of UTR sequence. Less sequence was recovered from the regulatory gene, myb111 (62%) likely due to its lower overall expression level and membership in a large gene family (see *Expression Analysis* below). One of the structural genes in the anthocyanin biosynthetic pathway, chalcone isomerase, did not pass the Blastn cut-off filter of E-value<10^−10^ likely due to previously reported elevated sequence divergence in *A. thaliana* [45] and the presence of at least one pseudogene in the *A. thaliana* genome [AT1G60290 [15]]. However, chalcone isomerase contigs were identified in the preceding step – the Velvet/Oases contig assembly. A previously characterized Sanger-sequenced version of this gene was appended to the transcriptome assembly in order to estimate expression differences for purple and white samples for all anthocyanin biosynthetic pathway loci.

**Table 2:** Transcriptome assembly results of anthocyanin biosynthetic pathway associated loci.

Locus^1^	*Parrya nudicaulis*CDS Length (bp)^2^	*Parrya nudicaulis*Assembly Length (bp)	Percent CDSCoverage	Additional 5′UTR (bp)	Additional 3′UTR (bp)
PAL1	2178	2035	93.4	0	25
CHS	1185	1185	100	54	175
F3H	1185	976	82.4	18	0
FLS	1011	1011	100	14	109
DFR	1128	1105	98.0	51	0
ANS	1173	1071	91.3	86	6
3GT	1350	1142	84.6	0	0
MYB111	1056	655	62.0	0	287
MEAN			89.0		

CDS = coding sequence.

1 Abbreviations are as follows: PAL1: phenylalanine ammonia-lyase 1, CHS: chalcone synthase, F3H: flavanone 3-hydroxylase, FLS: flavonol synthase, DFR: dihydroflavonol 4-reductase, ANS: anthocyanidin synthase, 3GT: UDP-glucose anthocyanidin 3-O-glucosyltransferase. Chalcone isomerase is not included since it did not have a significant BLAST hit E-value<10^−10^.

2 All coding sequence lengths except 3GT determined from cDNA sequences as in Dick et al. [13]. Coding sequence length for 3GT was based on alignment to *A. thaliana*.

### Expression Analysis

The *de novo* assembled transcriptome became the reference for remapping the reads to compare expression differences between purple and white samples from Savage River and 12 Mile Summit. Of the 86.35 million reads generated among the four samples, 37.27 million were aligned to the reference transcriptome (43.2%; Table S1 in File S1). A large portion of the generated reads was filtered-out because they had greater than two mismatches with the reference (41.53 million reads, 48.1%). Only a small portion of the aligned reads mapped to multiple locations (4.11 million reads; 4.8%), leaving 33.16 million reads that were uniquely aligned and used in the expression estimates (38.4%). The number of uniquely mapped reads used in the expression analysis was 6.29 and 6.96 million for the purple and white Savage River samples and 9.88 and 10.04 million for the purple and white 12 Mile Summit samples (Table S1 in File S1). The higher number of uniquely mapped reads in the 12 Mile Summit samples reflected the higher number of reads generated in these two samples due to improvements in the mRNA-Seq library prep during the nine months separating the Illumina runs of the two populations. In the end, the percent of uniquely mapped reads compared to the total number of reads generated ranged from 34.9% to 43.2%.

To avoid spurious comparisons of genes with very low expression, we removed 1,809 genes that had less than 10 unique reads mapped for any of the four samples. This removed nearly all genes with less than one RPKM (except three genes), a standard cutoff used for robust expression estimates of ∼2 kbp transcripts [29]. This left 10,986 genes in the transcriptome expression analysis. The length of the remaining genes ranged from 102 bp–3506 bp (median length = 676 bp). Expression estimates ranged from 0.779 to 14,720 RPKM.

The six core anthocyanin biosynthetic pathway enzyme coding genes had very high expression, all within the top 3.5% of genes in the transcriptome. CHS (purple) and dihydroflavonol 4-reductase were the most highly expressed anthocyanin biosynthetic pathway genes in the transcriptome falling within the top 0.21% and 0.39% of all genes identified in the transcriptome, respectively. Additional genes associated with the anthocyanin biosynthetic pathway (yet not typically considered core enzyme coding genes) including phenylalanine ammonia-lyase-1, flavonol synthase, and the regulatory locus, myb111, had much lower overall expression levels, ranking 8.4%, 35.9% and 71.9% in the transcriptome, respectively.

**Figure 3:**
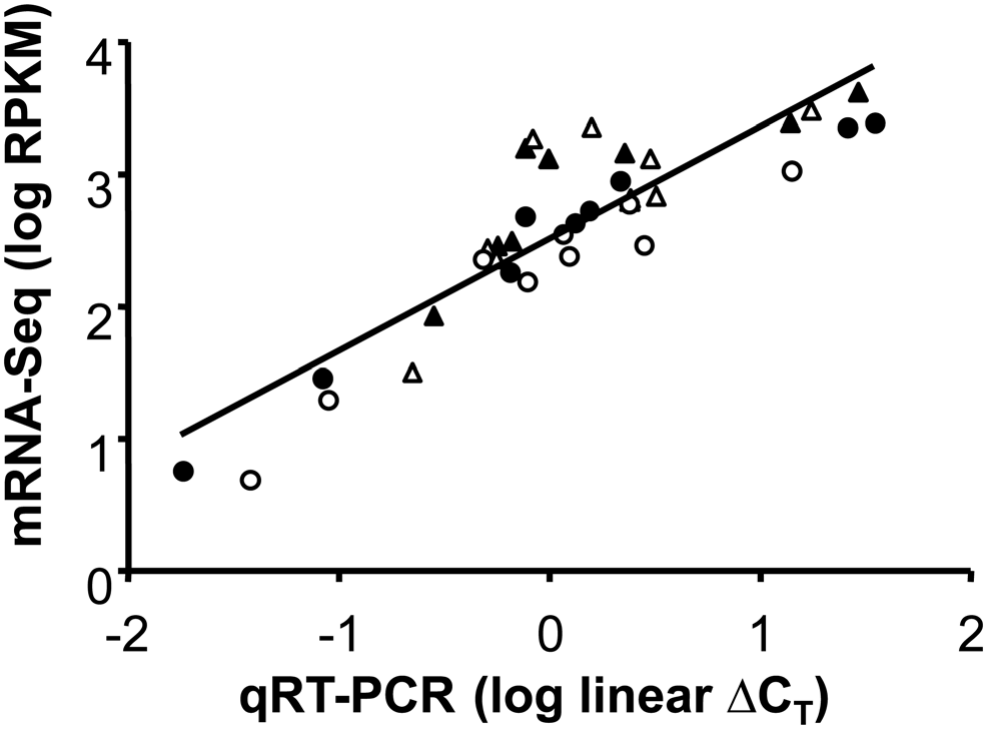
mRNA-Seq expression estimates validated with quantitative real time PCR (qRT-PCR) for anthocyanin biosynthetic pathway associated genes. mRNA-Seq expression estimates are strongly correlated with qRT-PCR for nine genes associated with the anthocyanin biosynthetic pathway for purple- and white-flowered samples from two populations – Savage River (filled triangles = purple petals; open triangles = white petals) and 12 Mile Summit (filled circles = purple petals; open circles = white petals). mRNA-Seq expression estimates are measured in reads per kilobase exon per million uniquely mapped reads (RPKM) and log transformed to fit assumptions of normality. The qRT-PCR data, measured using the ΔC_T_ method, have been linearized and log transformed for comparison to the Illumina expression estimates.

**Figure 4:**
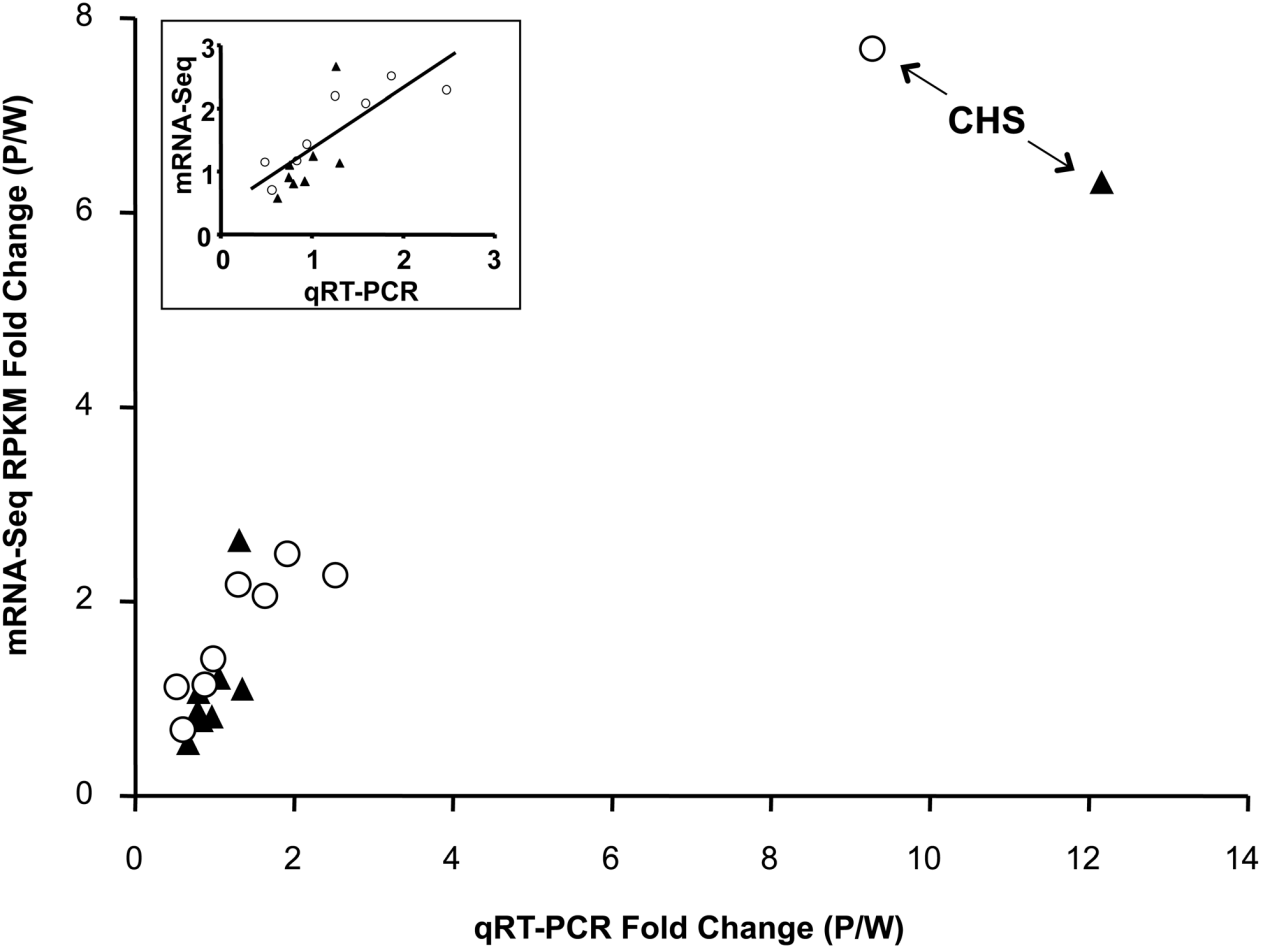
Purple/white fold-change comparison of mRNA-Seq and quantitative real time PCR (qRT-PCR) expression estimates. For both methods, chalcone synthase (CHS) had the greatest expression difference between purple- and white-flowered samples among nine genes in the anthocyanin biosynthetic pathway (Savage River = filled triangles; 12 Mile Summit = open circles). The remaining anthocyanin biosynthetic pathway genes have comparable expression between purple- and white-flowered samples and show a significant correlation between mRNA-Seq and qRT-PCR expression estimates (inset).

We used these six anthocyanin biosynthetic pathway core enzyme coding genes and three genes associated with the anthocyanin biosynthetic pathway (described above) to test for correlations between expression estimated from mRNA-Seq data and previous expression estimates from qRT-PCR. Although the expression levels of these nine genes span a large range (5–4,285 RPKM), we found a very strong correlation for the same four RNA samples in qRT-PCR [log(RPKM)  = 0.84*log(linear ΔC_T_)+2.53; r^2^ = 0.78, F_1,35_ = 119.89, *P*<0.0001; Fig. 3). Furthermore, CHS has the largest P/W fold-change in expression using both methods (Fig. 4), but the P/W fold-change from the mRNA-Seq data is lower (P/W_SavageRiver_ = 6.35; P/W_12MileSummit_ = 7.72) than the fold-change estimated from qRT-PCR (P/W_SavageRiver_ = 12.09; P/W_12MileSummit_ = 9.23). After removing CHS as an outlier, there was still a significant correlation between mRNA-Seq and qRT-PCR estimates of the P/W fold-change (qRT-PCR Fold-change = 0.60*RPKM Fold-change+0.23; r^2^ = 0.61, F_1,15_ = 21.84, *P* = 0.0004; Fig. 4 inset).

**Figure 5:**
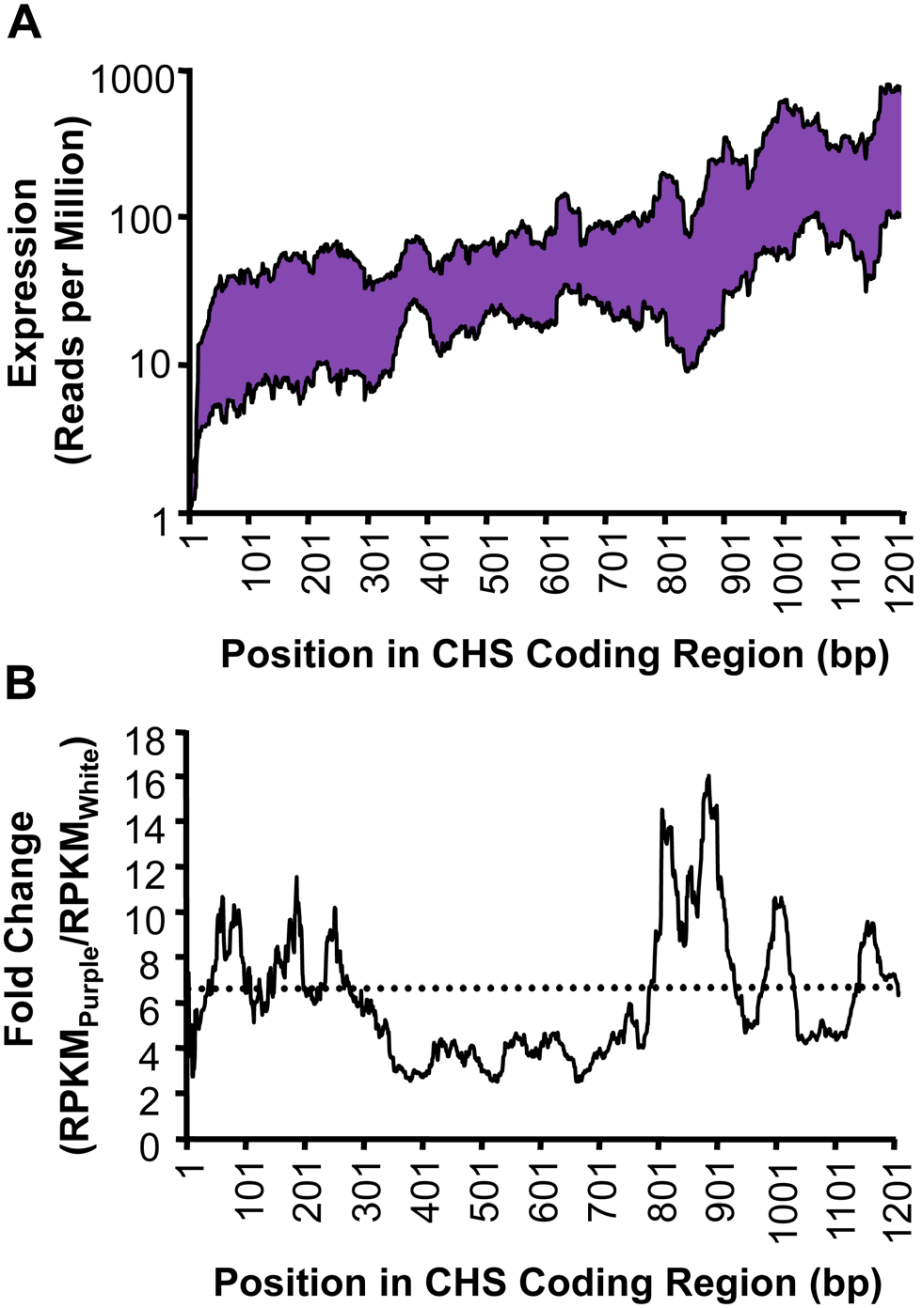
Expression profile for chalcone synthase (CHS). (A) mRNA-Seq detects substantially higher expression in purple than in white Savage River samples along the entire coding region of CHS. Some 3′-bias is apparent from the increased expression estimates towards the 3′ end of the coding region. (B) A comparison of the purple/white fold-change along the entire coding region of CHS indicates no consistent effect of the 3′-bias on the ratio of purple to white expression. The average fold-change across the gene for the Savage River samples is indicated with a dotted line (P/W = 6.35).

When examining read density across the coding region for CHS, we detected the expected 3′ bias in all four samples based on the poly-A+ RNA based libraries [Savage River samples depicted in Fig. 5A [30]], yet the fold-change comparison across the coding region showed no consistent trend (Fig. 5B). The middle 500 bp of the coding region has lower than average expression differences and the 5′ and 3′ ends have much more erratic P/W estimates (Fig. 5B).

**Figure 6:**
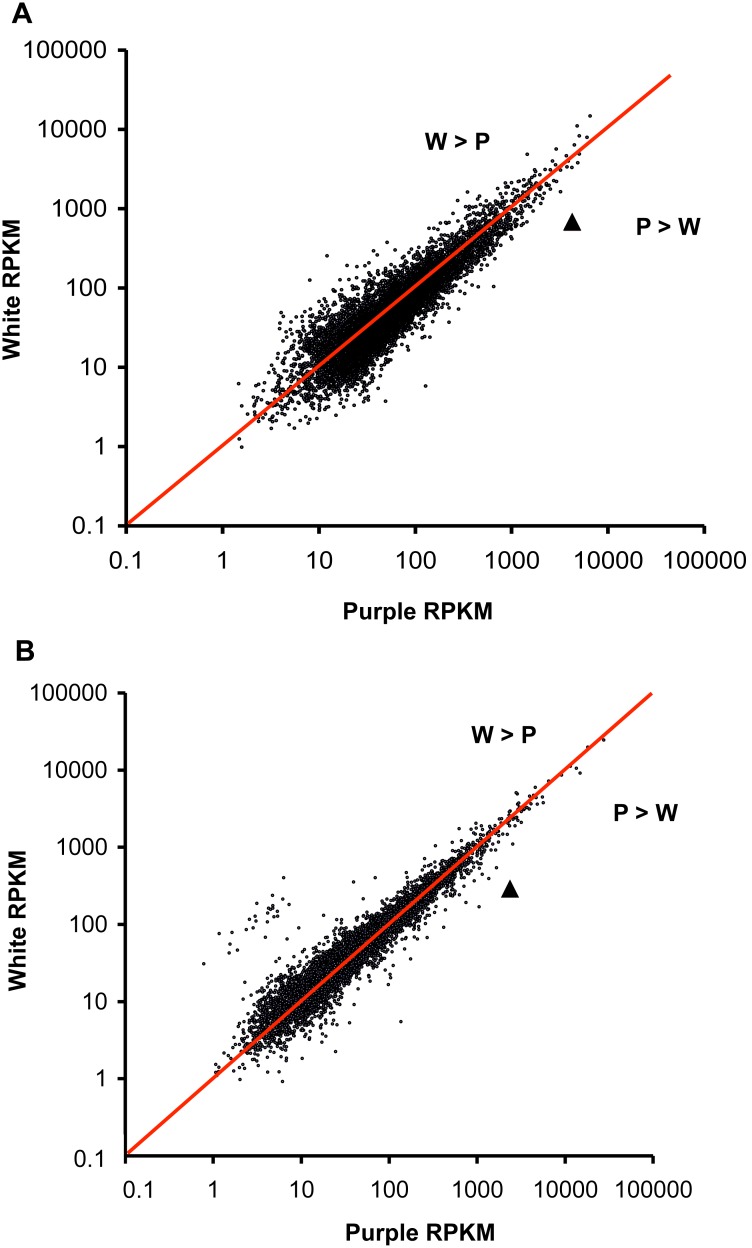
Transcriptome-wide comparison of purple and white expression. Expression estimates are measured in reads per kilobase exon per million uniquely mapped reads (RPKM). The red line indicates equal expression in purple and white samples. Chalcone synthase is indicated with a triangle. (A) Savage River samples; (B) 12 Mile Summit samples.

To identify individual genes with consistently different expression between purple and white petals, we examined the results as two pairs of purple-white samples (Savage River samples depicted in Fig. 6A and 12 Mile Summit samples depicted in Fig. 6B). We then applied a consistency cutoff of ±0.25 (Consistency = RelativeDifference_SavageRiver_ – RelativeDifference_12MileSummit_), allowing us to focus on genes with the most consistent relative expression differences between purple and white. For the Savage River purple versus white comparison, 10.9% of the 1,405 differentially expressed genes (genes with RD>0.5 or RD<–0.5) fell within our consistency cut-off. For the 12 Mile Summit comparison, 22.3% of the 557 differentially expressed genes were within the consistency cut-off. Using the average RD from both population comparisons, 59.1% of the 208 differentially expressed genes fell within our (arbitrary) threshold for consistency.

**Figure 7:**
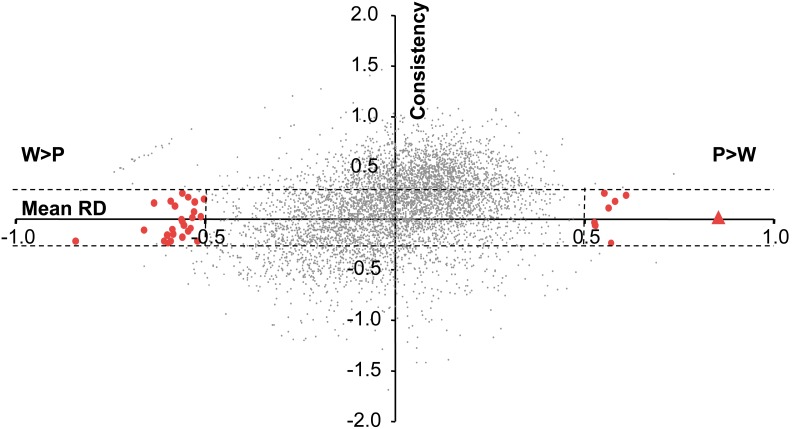
Identification of consistently differentially expressed genes. Consistently differentially expressed genes were identified by comparing the mean relative difference (RD) between purple- and white-flowered samples from Savage River and 12 Mile Summit to the consistency of the RD estimates between those two populations. RD was calculated for each purple-white pair of samples at a population [RD = (purple RPKM – white RPKM)/max(purple RPKM, white RPKM)]. Consistency is the difference between RD Savage River and RD 12 Mile Summit. We focused on points with RD>0.5 or RD<–0.5 and with consistency less than 0.25 and greater than –0.25 (larger red filled circles). Chalcone synthase is indicated with a red triangle as the most consistently differentially expressed gene with greater expression in purple than white. There are approximately three times more consistently differentially expressed genes with white expression greater than purple (25 genes) compared to the number of consistently differentially expressed genes with purple greater than white (8 genes).

Among the 4,593 consistently expressed genes, CHS had the largest relative difference when examining genes where purple expression was greater than white (P/W average = 7.03; Fig. 7). Although there were seven other genes that were consistently differentially expressed with P>W, the fold-change was considerably smaller than that observed for CHS (remaining seven genes P/W ranged from 2.14–2.80; Table S2 in File S1). On the opposite end of the spectrum, there were 25 genes that consistently exhibited W>P (Fig. 7; Table S3 in File S1), none of which have been previously associated with the anthocyanin biosynthetic pathway [46]. Two of the three most differentially expressed genes with W>P were unknown proteins (AT1G10020.1, AT3G01950.1 with W/P = 3.04, and 2.85, respectively). Twenty of the remaining 22 genes with consistent W>P expression differences blast to known genes in *A. thaliana*.

### Validation of Consistently Differentially Expressed Genes

**Figure 8:**
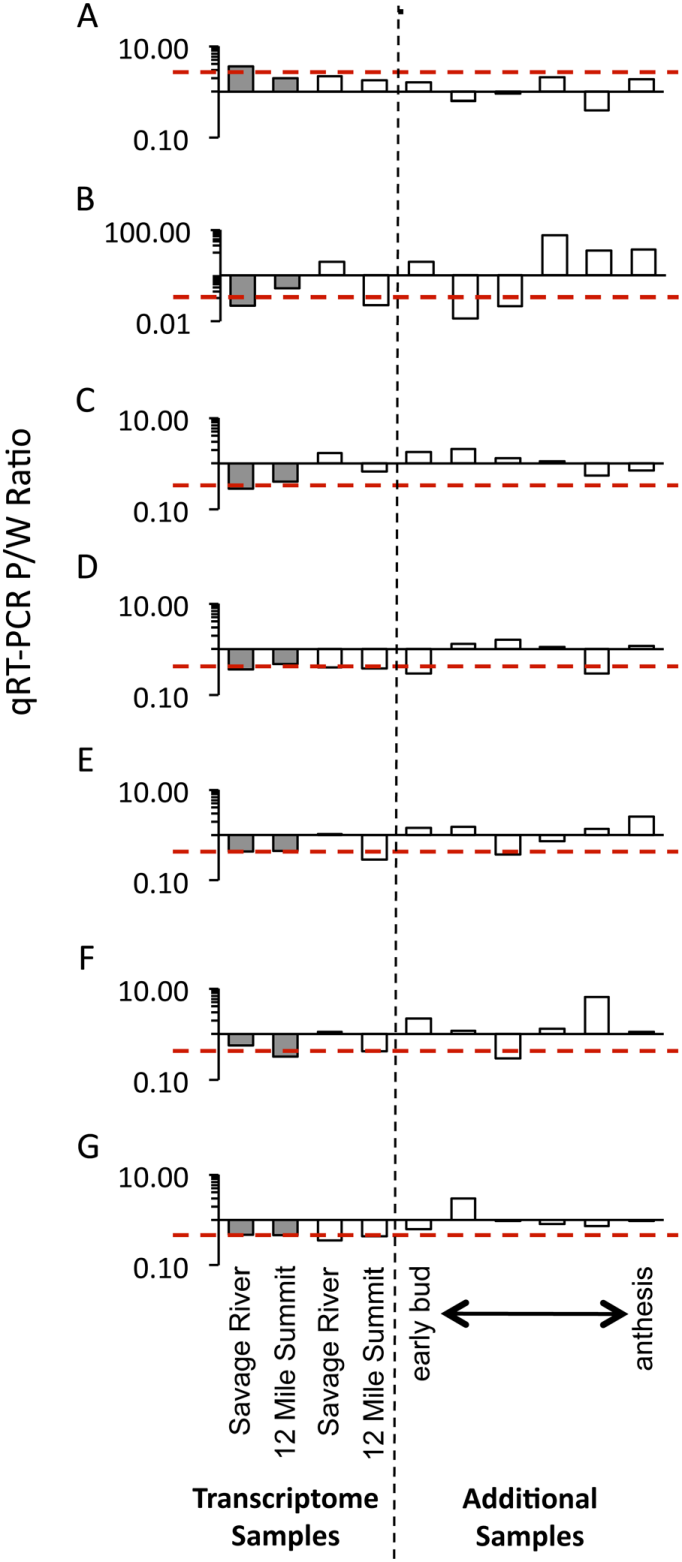
Quantitative Real Time PCR (qRT-PCR) survey of targeted consistently differentially expressed genes. Seven genes from the transcriptome analysis were selected for a qRT-PCR survey across a broader set of RNA samples (see Tables S2 and S3 in File S1 for expression results for these genes). Based on mRNA-Seq results, we selected the second highest differentially expressed gene with greater expression in purple compared to white (GDSL-motif lipase/hydrolase family; AT5G45950 depicted in A.) and six genes with consistently higher expression in white petals [B. 7SL RNA gene (AT2G31141); C. unknown protein (AT1G10020); D. CDSP32 (cp drought-induced 32 kD stress-induced protein; AT1G76080); E. EXL5 (exordium like 5; AT2G17230); F. ERD5 (early responsive to dehydration 5; AT3G30775); G. DND1 (defense no death 1; AT5G15410)]. Each bar represents the P/W ratio for a pooled sample at that developmental stage. mRNA-Seq based expression estimates from Savage River (early bud) and 12 Mile Summit (late bud) are represented with filled grey bars. The mean expression difference from the transcriptome analysis of these two populations is indicated with a red dashed line. The same samples used in mRNA-Seq were validated with qRT-PCR depicted as the white filled bars to the left of the vertical dashed line. Six additional P/W comparisons were made across developmental stages (white bars to the right of the vertical dashed line).

We conducted TaqMan qRT-PCR assays on seven consistently differentially expressed genes (Tables S2 and S3 in File S1) for the original four pooled samples used in the transcriptome analysis and an additional eight samples. As expected, the gene from the transcriptome analysis with the second highest P/W ratio (GDSL-motif lipase/hydrolase family protein, AT5G45950) had higher qRT-PCR-based expression in the purple-flowered sample from both Savage River and 12 Mile Summit (average P/W = 1.96; Fig. 8). For the six W>P genes examined with qRT-PCR, the 12 Mile Summit pair of samples had consistently higher expression in white petals compared to purple as expected from mRNA-Seq analyses (Fig. 8). However, for the Savage River pair of samples, only two of these six genes had greater expression in the white-flowered sample (a drought stress gene, AT1G76080, and a defense gene, AT5G15410), two genes had similar expression between the two color morphs (an early response to dehydration gene, AT3G30775, and a gene with unknown function, EXL5, AT2G17230), and the last two had greater expression in the purple-flowered sample (a 7SL RNA gene, AT2G31141, and the unknown gene, AT1G10020), contrary to the results from the mRNA-Seq analysis.

After expanding the sampling of purple and white petal samples from four to a total of 12, we found no consistently differentially expressed genes across a range of floral developmental stages (Fig. 8). Differential expression was most consistent between the Illumina samples and the broader sampling for the pathogen defense gene DND1 (AT5G15410 with five out of six additional cDNA samples having W>P expression). Three of the additional samples were from bud-stage petals and still show substantial deviations from the expression levels predicted by mRNA-Seq, suggesting that variation is not due to changes arising in later developmental stages. The lack of consistency could reflect our pursuit of false positives or be caused by variation among individuals sampled at different developmental stages.

### Gene Ontology Analysis

We tested for significant enrichment in GO terms among the top 2% of consistently differentially expressed genes between the two populations (N = 92 genes). For P>W, we found eight terms enriched at the *P*<0.01 level and an additional 26 terms enriched at the *P*<0.05 level. Significantly enriched gene ontology terms represented by more than one gene include “response to abiotic stimulus” (six genes; *P* = 0.016) and “response to radiation” (3 genes; *P* = 0.048). Thirty of the 92 genes in the top 2% of consistently differentially expressed genes were unannotated and therefore not included in the GO analysis.

Among the top 2% of genes with consistently higher white expression than purple, there were 18 significant terms (*P*<0.01) and 42 marginally significant terms (*P*<0.05) exhibiting enrichment. There were ten genes in the general category “response to stress” (*P* = 0.0049) which includes sub-categories such as “response to cold” (three genes; *P* = 0.009), “plant-type hypersensitive response” (i.e. pathogen response; two genes; *P* = 0.002), “innate immune response” (three genes; *P* = 0.020) and “response to water deprivation” (two genes; *P* = 0.032). Another 28 of the 92 genes in the top 2% of consistently differentiated genes with W>P were unannotated and therefore not included in this analysis.

## Discussion

### Expression of Anthocyanin Biosynthetic Pathway Candidate Genes

Expression analysis of the petal bud transcriptome clearly indicates that CHS has the greatest expression difference between purple and white petal buds and is the top expression candidate for the potential cause of white petals in *P. nudicaulis* [13]. Among the ∼10,000 genes assayed for expression in the petal, CHS had the largest P>W fold-change difference, more than 2.5× greater than the next most differentially expressed gene with P>W (Table S2 in File S1). The strong correlation between expression estimated by mRNA-Seq and qRT-PCR for anthocyanin biosynthetic pathway associated genes validated our *de novo* transcriptome assembly and expression estimates. However, the average P/W ratio for CHS was consistently lower for mRNA-Seq (mean P/W = 7.03×) than that previously documented for qRT-PCR (mean P/W = 10.66×). This discrepancy was unlikely caused by the small region assayed by qRT-PCR since the mean mRNA-Seq P/W fold-change estimated across the 78 bp length of the qPCR-amplified region is very similar to the expression estimated for the entire gene by mRNA-Seq (P/W = 7.70×; Fig. 5). Furthermore, it is unlikely that the discrepancy in CHS fold-change is due to inherent errors in the mRNA-Seq methodology or bioinformatics pipeline since most anthocyanin biosynthetic pathway associated genes show higher expression in mRNA-Seq when compared to qRT-PCR (Fig. 4).

Tissue-specific downregulation of a single gene at the entry point to the anthocyanin biosynthetic pathway is rare among angiosperms in which the loss of floral anthocyanins has been characterized [4], [47], [48]. Most cases of natural, tissue-specific loss-of-floral anthocyanins involve downregulation of multiple anthocyanin biosynthetic pathway associated loci often caused by R2R3 myb regulatory elements [4], [47], [49]. Transcriptome analysis of *P. nudicaulis* identified 53 mybs expressed in petal buds compared to the 126 mybs in the entire *A. thaliana* genome [50]. We did not assemble any of the known tissue specific regulators of the anthocyanin biosynthetic pathway [Atmyb75, Atmyb90, Atmyb113 and Atmyb114; [50], [51]] and the majority of the mybs assembled had very low expression in *P. nudicaulis* (median expression = 35.01 RPKM). For Atmyb111, a known regulator of CHS along with chalcone isomerase, flavanone 3-hydroxylase, flavonol synthase and UDP-glucose anthocyanidin 3-O-glucosyltransferase in *A. thaliana* cotyledons [51], we confirmed the absence of any consistent differences in expression between purple and white petals in *P. nudicaulis* (P/W = 0.87). Since expression of Atmyb111 is unlikely the cause of the CHS downregulation, we looked more broadly at the ten most highly expressed mybs in the *P. nudicaulis* petal transcriptome (greater than 80 RPKM). Only two mybs show consistent differential expression between purple and white petals in the two populations surveyed in this study (Atmyb32 and Atmyb4). These two mybs are closely related and essential regulators for flavonoid production, a key component to pollen viability in *A. thaliana* [50], [52]. Furthermore, overexpression of Atmyb4 downregulates CHS along with two upstream genes, 4-coumarate-CoA ligase 3 and 4-coumarate-CoA ligase 1 in *A. thaliana* [52]. In *P. nudicaulis*, both of these mybs have W>P (mean W/P = 1.6× and 1.3×), yet we find no correlated expression differences between purple and white petals for the other two regulatory targets in *A. thaliana*: 4-coumarate-CoA ligase 3 and 4-coumarate-CoA ligase 1 (mean P/W = 1.2 for both genes in *P. nudicaulis*). Future studies examining sequence differences in these mybs are necessary to assess any involvement they may have in the purple-white color difference in *P. nudicaulis*.

### Expression of Non-Anthocyanin Biosynthetic Pathway Related Genes

Transcriptome-wide expression analysis revealed a much larger set of genes that are consistently differentially expressed where W>P. None of these genes are immediately associated with the anthocyanin biosynthetic pathway across a diversity of angiosperms [15], [17], [18], [19], nor have they been identified in the broader metabolic network of *A. thaliana* [18]. Differential expression of these genes is unlikely due to co-expression of genes physically adjacent to CHS [53] since only six of the 25 genes with W>P are on the same chromosome as CHS (chromosome five) and the closest gene is greater than 515 kbp from CHS (DND1, AT5G15410.2) in *A. thaliana*. Genome wide coexpression of neighboring genes in *A. thaliana* was undetectable beyond 12 kbp [53] and is weak once separated by two or more intervening genes [54].

One hypothesis is that these consistently differentially expressed genes with W>P represent a physiological consequence of the loss-of-petal anthocyanins in white flowers in response to increased sensitivity to petal-specific abiotic stress. Of the 25 consistently differentially expressed W>P genes, 20 were probed in a collection of nine *A. thaliana* microarray experiments primarily involving abiotic stress-induction [17]. Sixteen genes were correlated with CHS at least once, but none of these genes were correlated in more than three stress experiments. In one particular cold stress experiment (GEO Accession: GSE6177), four genes in our W>P list had correlated expression with CHS (*P*<0.05). Several of these genes have known functions in cold and drought stress consistent with the stress-related function of anthocyanins. Species with floral anthocyanin polymorphisms have demonstrated a decreased performance (and sometimes decreased fitness) of white morphs under stressful environments [e.g., heat, drought, herbivory, pathogen attack [4], [7], [8]], even when the loss of anthocyanins is restricted to the flower petals, like in *P. nudicaulis*. We hypothesize that the transcriptional consequences documented here compensate for the loss of petal anthocyanins in these more benign southern populations, but may not be sufficient to do the same in more northern populations leading to the recently documented selection against lighter colored morphs and the exclusion of white flowered individuals along Alaska’s Arctic Coastal Plain [13], [14]. Additional field and molecular studies are necessary to test this hypothesis.

A qRT-PCR survey of additional individuals for seven genes with W>P revealed that these genes are not universally differentially expressed in white-flowered individuals of these populations. Either (1) the low expression levels of most of these genes causes dramatic fluctuations in P/W ratio by chance alone producing false positives (four out of six genes had less than 100 RPKM), or (2) the inconsistent stress-response level in white-petals (or dampened response in purple-petals) is not universal and may be dependent on the individual’s specific microenvironment. When we rank the genes in this survey by their overall expression levels, we see no trend in the consistency of W>P suggesting that low expression alone is not the primary cause of the variation among samples. We conclude that the increased sensitivity of white-flowered individuals as detected in their transcriptional response is microenvironment-dependent. Controlled manipulative experiments that induce a consistent stress-response on both color morphs will be necessary to determine if these are false positives or if these genes with W>P are a consequence of the loss of petal anthocyanins.

### Methodological Concerns

The *de novo* transcriptome assembly pipeline developed for *P. nudicaulis* captured sequences for nearly half of the coding sequence of ∼13,000 genes [approximately half of the known genes in *A. thaliana*; [15]], yet our method has some limitations. First, spurious expression differences producing false positives can easily arise when relying on few samples, especially when repeatedly examining fold-changes for genes with low-expression (53.2% of the genes surveyed for expression have less than 50 RPKM). For example, if we only used one pair of samples, we would have falsely inferred several genes involved in cell wall biosynthesis were differentially expressed (see 27 genes with W>P in Fig. 6B that are not differentially expressed in Fig. 6A; also visible in the upper left quadrant of Fig. 7). We have reduced the probability of spurious fold-change estimates by pooling individuals of the same developmental stage and petal color into a single sample, including pooled samples from geographically distinct populations, and removing all genes with less than 10 mapped reads. Our sampling design was not ideal for drawing robust statistical conclusions, but rather was designed to identify candidate genes and develop hypotheses for future exploration.

Second, by using a very stringent cut-off for the blastn and reciprocal best-hit nucleotide blast analyses (E-value<10^−10^) to increase the probability of correct homolog identification, we compromised our ability to identify genes that have high divergence from *A. thaliana*. For instance, several contigs for CHI were assembled *de novo*, but the elevated coding region sequence divergence (22.5% compared to 11% for remaining anthocyanin biosynthetic pathway genes) failed to meet our blastn threshold. Yet even in this case, chalcone isomerase still appears to be under strong purifying selection compared to *A. thaliana* (Ka/Ks = 0.098). In general, using stringent blastn and reciprocal best-hit nucleotide blast cut-offs leads to an underestimate of the petal transcriptome size and removes genes with elevated rates of molecular evolution in comparison to *A. thaliana*. Although sequence divergence and expression divergence can be correlated [[55], but see exceptions [56], [57]], this unlikely biased our P/W comparison within *P. nudicaulis*.

A third limitation of *de novo* transcriptome assembly is discerning closely-related paralogs that are simultaneously expressed in the same tissue [25]. Paralogs complicate the contig assembly and expression estimates, yet can be partly accommodated using analytical methods that are optimized for heterogeneous datasets [e.g., Velvet/Oases; [39]]. In attempts to reduce the assembly of paralogous sequences, we included a reciprocal-best-hit blastn step into our *de novo* assembly pipeline using a stringent E-value threshold. Even with this procedure, we identified 566 alignments with sequence variation greater than that expected in natural populations. Upon visual examination of these alignments, we identified numerous paralogs with similar coding regions, yet highly divergent untranslated regions. We considered these together in the expression analysis since their function is inferred to be similar based on their nearly identical coding regions and coexpression in the same tissue. We also confirmed each of the consistently differentially expressed genes (Tables S2 and S3 in File S1) using only purple and only white contigs to form the reference for expression analysis in case the two color morphs were expressing different paralogs. There was no consistent bias in the P/W fold-change estimates based on the sequences that were used for the reference sequence (likely ameliorated by the accommodation of 2–4 mismatches by Mosaik). Future studies employing longer read-lengths (greater than 200 bp including paired-end reads) can improve the chances of detecting paralogs assuming they are sufficiently differentiated at the sequence level.

Polyploidy can also complicate transcriptome analysis by creating genome-wide paralogy and plants of high northern latitudes are renown for the high frequency of polyploids [58]. In particular, *P. nudicaulis* has been reported to have diploid (2n = 14) and tetraploid populations [2n = 28 [59]], yet we found no evidence of polyploidy within the Alaskan populations studied here. The diploid genome size reported here (2C = 2.08) is very similar to twice the haploid genome estimate of *P. nudicaulis* (1C = 1.08 pg) with diploid chromosome counts of 2n = 14 [35]. Although the *P. nudicaulis* genome is over six times larger than the *A. thaliana* genome [1C = 0.16 pg [60]], it is only ∼2× larger than the inferred ancestral state for the Brassicaceae [1C = 0.504 pg [35]]. In a previous study [13], we used degenerate primers designed to amplify the anthocyanin biosynthetic pathway associated genes across angiosperms. After sequencing more than 12 clones per RT-PCR, we consistently found the same alleles expressed in petals and leaves with no evidence of paralogy. This, in combination with a genomic DNA survey of the anthocyanin biosynthetic pathway loci, which included sequence comparisons of intron and/or untranslated regions (which are most likely to reveal young paralogs), showed no evidence of recent genome wide duplication [13].

## Conclusions

Transcriptome analysis is consistent with a previous study suggesting the loss of petal anthocyanins in *P. nudicaulis* is correlated with downregulation of CHS. We documented 3× more genes with consistently greater expression in white petals than in purple petals, many of which are involved in stress-response consistent with the numerous non-pollinator roles of floral anthocyanins. However, the physiological and ecological consequences of the loss of floral anthocyanins may be environment-dependent and require additional controlled stress experiments to confirm the cause of these consistently differentially expressed genes with higher expression in white petals compared to purple petals.

## Supporting Information

File S1 **Supporting tables.** Table S1, Fate of reads generated during the transcriptome assembly and expression analysis. Table S2, Genes with consistently higher expression in purple compared to white petal samples. Table S3, Genes with consistently higher expression in white compared to purple petal samples.
(DOCX)

## References

[1] Bradshaw HD, Schemske DW (2003) Allele substitution at a flower colour locus produces a pollinator shift in monkeyflowers. Nature 426: 176–178. 14614505 10.1038/nature02106

[2] Fenster CB, Armbruster WS, Wilson P, Dudash MR, Thompson JD (2004) Pollination syndromes and floral specialization. Annual Review of Ecology and Systematics 35: 375–403.

[3] Melendez-Ackerman E, Campbell DR (1998) Adaptive significance of flower color and inter-trait correlations in an *Ipomopsis* hybrid zone. Evolution 52: 1293–1303. 28565388 10.1111/j.1558-5646.1998.tb02011.x

[4] Rausher MD (2008) Evolutionary transitions in floral color. International Journal of Plant Sciences 169: 7–21.

[5] Streisfeld M, Kohn JR (2005) Contrasting patterns of floral and molecular variation across a cline in *Mimulus aurantiacus* . Evolution 59: 2548–2559. 16526503

[6] Whittall JB, Hodges SA (2007) Pollinator shifts drive increasingly long nectar spurs in columbine flowers. Nature 447: 706–712. 17554306 10.1038/nature05857

[7] Strauss SY, Whittall JB (2006) Non-pollinator agents of selection on floral traits. In: Harder LD, Barrett SCH, editors. Ecology and evolution of flowers. Oxford: Oxford University Press. 120–138.

[8] von Wettberg E, Stanton ML, Whittall JB (2010) How anthocyanin mutants respond to stress: the need to distinguish between stress tolerance and maximal vigour. Evolutionary Ecology Research 12: 457–476.

[9] Winkel-Shirley B (2001) Flavonoid biosynthesis. A colorful model for genetics, biochemistry, cell biology, and biotechnology. Plant Physiology 126: 485–493. 11402179 10.1104/pp.126.2.485 PMC1540115

[10] Mosquin T (1966) Reproductive specialization as a factor in the evolution of the Canadian flora. In: Taylor RJ, Ludwig RA, editors. The evolution of Canada’s flora. Toronto, Canada: University of Toronto Press. 41–63.

[11] Pielou EC (1994) A naturalist’s guide to the Arctic. Chicago, IL: University of Chicago Press. 329 p.

[12] Whittall JB, Carlson ML (2009) Plant defense: a pre-adaptation for pollinator shifts. New Phytologist 182: 5–8. 19291069 10.1111/j.1469-8137.2009.02796.x

[13] Dick C, Buenrostro J, Butler T, Carlson ML, Kliebenstein DJ, et al (2011) Arctic mustard flower color polymorphism controlled by petal-specific downregulation at the threshold of the anthocyanin biosynthetic pathway. PLoS ONE 6: e18230. 21490971 10.1371/journal.pone.0018230 PMC3072389

[14] Fulkerson JR, Whittall JB, Carlson ML (2012) Reproductive ecology and severe pollen limitation in the polychromic tundra plant, *Parrya nudicaulis* (Brassicaceae). PLoS ONE 7: e32790. 22427886 10.1371/journal.pone.0032790 PMC3299698

[15] Swarbreck D, Wilks C, Lamesch P, Berardini TZ, Garcia-Hernandez M, et al (2008) The *Arabidopsis* Information Resource (TAIR): gene structure and function annotation. Nucleic Acids Research 36: D1009–D1014. 17986450 10.1093/nar/gkm965 PMC2238962

[16] Martin D, Brun C, Remy E, Mouren P, Thieffry D, et al (2004) GOToolBox: functional analysis of gene datasets based on gene ontology. Genome Biology 5: R101. 15575967 10.1186/gb-2004-5-12-r101 PMC545796

[17] Obayashi T, Hayashi S, Saeki M, Ohta H, Kinoshita K (2009) ATTED-II provides coexpressed gene networks for *Arabidopsis* . Nucleic Acids Research 37: D987–D991. 18953027 10.1093/nar/gkn807 PMC2686564

[18] Lee I, Ambaru B, Thakkar P, Marcotte EM, Rhee SY (2010) Rational association of genes with traits using a genome-scale gene network for *Arabidopsis thaliana* . Nat Biotech 28: 149–156. 10.1038/nbt.1603 PMC2857375 20118918

[19] Manfield IW, Jen C-H, Pinney JW, Michalopoulos I, Bradford JR, et al (2006) *Arabidopsis* Co-expression Tool (ACT): web server tools for microarray-based gene expression analysis. Nucleic Acids Research 34: W504–W509. 16845059 10.1093/nar/gkl204 PMC1538833

[20] Adams MD, Kelley JM, Gocayne JD, Dubnick M, Polymeropoulos MH, et al (1991) Complementary DNA sequencing: expressed sequence tags and human genome project. Science 252: 1651–1656. 2047873 10.1126/science.2047873

[21] Caron H, van Schaik B, van der Mee M, Baas F, Riggins G, et al (2001) The human transcriptome map: clustering of highly expressed genes in chromosomal domains. Science 291: 1289–1292. 11181992 10.1126/science.1056794

[22] Lee NH, Weinstock KG, Kirkness EF, Earle-Hughes JA, Fuldner RA, et al (1995) Comparative expressed-sequence-tag analysis of differential gene expression profiles in PC-12 cells before and after nerve growth factor treatment. Proceedings of the National Academy of Sciences 92: 8303–8307. 10.1073/pnas.92.18.8303 PMC41145 7667285

[23] Velculescu VE, Zhang L, Vogelstein B, Kinzler KW (1995) Serial analysis of gene expression. Science 270: 484–487. 7570003 10.1126/science.270.5235.484

[24] Gibbons JG, Janson EM, Hittinger CT, Johnston M, Abbot P, et al (2009) Benchmarking next-generation transcriptome sequencing for functional and evolutionary genomics. Molecular Biology and Evolution 26: 2731–2744. 19706727 10.1093/molbev/msp188

[25] Renaut S, Nolte AW, Bernatchez L (2010) Mining transcriptome sequences towards identifying adaptive single nucleotide polymorphisms in lake whitefish species pairs (*Coregonus* spp. Salmonidae). Molecular Ecology 19: 115–131. 20331775 10.1111/j.1365-294X.2009.04477.x

[26] Rokas A, Abbot P (2009) Harnessing genomics for evolutionary insights. Trends in Ecology and Evolution 24: 192–200. 19201503 10.1016/j.tree.2008.11.004

[27] Wang XW, Luan JB, Li JM, Bao YY, Zhang CX, et al (2010) *De novo* characterization of a whitefly transcriptome and analysis of its gene expression during development. BMC Genomics 11: 400–410. 20573269 10.1186/1471-2164-11-400 PMC2898760

[28] Mizuno H, Kawahara Y, Sakai H, Kanamori H, Wakimoto H, et al (2010) Massive parallel sequencing of mRNA in identification of unannotated salinity stress-inducible transcripts in rice (*Oryza sativa* L.). BMC Genomics 11: 683. 21122150 10.1186/1471-2164-11-683 PMC3016417

[29] Mortazavi A, Williams BA, McCue K, Schaeffer L, Wold B (2008) Mapping and quantifying mammalian transcriptomes by RNA-Seq. Nature Methods 5: 621–628. 18516045 10.1038/nmeth.1226 PMC13303166

[30] Nagalakshmi U, Wang Z, Waern K, Shou C, Raha D, et al (2008) The transcriptional landscape of the yeast genome defined by RNA sequencing. Science 320: 1344–1349. 18451266 10.1126/science.1158441 PMC2951732

[31] Pan Q, Shai O, Lee LJ, Frey BJ, Blencowe BJ (2008) Deep surveying of alternative splicing complexity in the human transcriptome by high-throughput sequencing. Nat Genet 40: 1413–1415. 18978789 10.1038/ng.259

[32] Wilhelm BT, Marguerat S, Watt S, Schubert F, Wood V, et al (2008) Dynamic repertoire of a eukaryotic transcriptome surveyed at single-nucleotide resolution. Nature 453: 1239–1243. 18488015 10.1038/nature07002

[33] Gilad Y, Pritchard JK, Thornton K (2009) Characterizing natural variation using next-generation sequencing technologies. Trends in Genetics 25: 463–471. 19801172 10.1016/j.tig.2009.09.003 PMC3994700

[34] Dolezel J, Greilhuber J, Suda J (2007) Estimation of nuclear DNA content in plants using flow cytometry. Nat Protocols 2: 2233–2244. 17853881 10.1038/nprot.2007.310

[35] Lysak MA, Koch MA, Beaulieu JM, Meister A, Leitch IJ (2009) The dynamic ups and downs of genome size evolution in Brassicaceae. Molecular Biology and Evolution 26: 85–98. 18842687 10.1093/molbev/msn223

[36] Doležel J, Bartoš J, Voglmayr H, Greilhuber J (2003) Nuclear DNA content and genome size of trout and human. Cytometry Part A 51A: 127–128. 10.1002/cyto.a.10013 12541287

[37] Bustin SA, Benes V, Garson JA, Hellemans J, Huggett J, et al (2009) The MIQE Guidelines: Minimum information for publication of quantitative real-time PCR experiments. Clinical Chemistry 55: 611–622. 19246619 10.1373/clinchem.2008.112797

[38] Zerbino D, Birney D (2008) Velvet: Algorithms for *de novo* short read assembly using De Brujin graphs. Genome Research 18: 821–829. 18349386 10.1101/gr.074492.107 PMC2336801

[39] Zerbino DR (2010) Using the Velvet *de novo* assembler for short read sequencing technologies. Current Protocols in Bioinformatics 31: 11.15.1–11.15.12. 10.1002/0471250953.bi1105s31 PMC2952100 20836074

[40] Pruitt KD, Tatusova T, Maglott DR (2006) NCBI reference sequences (RefSeq): A curated non-redundant sequence database of genomes, transcripts and proteins. Nucleic Acids Research 35: D61–D65. 17130148 10.1093/nar/gkl842 PMC1716718

[41] Hirsh AE, Fraser HB (2001) Protein dispensability and rate of evolution. Nature 411: 1046–1049. 11429604 10.1038/35082561

[42] Edgar RC (2004) MUSCLE: Multiple sequence alignment with high accuracy and high throughput. Nucleic Acids Research 32: 1792–1797. 15034147 10.1093/nar/gkh340 PMC390337

[43] Livak KJ, Schmittgen TD (2001) Analysis of relative gene expression data using real-time quantitative PCR and the 2^−ΔΔCt^ method. Methods 25: 402–408. 11846609 10.1006/meth.2001.1262

[44] Bennett M, Leitch IJ (2005) Plant DNA C-values database. Royal Botanic Gardens, Kew, UK. Plant DNA C-values Database website. Available: http://www.kew.org/cvalues/. Accessed 2014 June 22.

[45] Ramos-Onsins SE, Puerma E, Balana-Alcaide D, Salguero D, Aguade M (2008) Multilocus analysis of variation using a large empirical data set: Phenylpropanoid pathway genes in *Arabidopsis thaliana* . Molecular Ecology 17: 1211–1223. 18221273 10.1111/j.1365-294X.2007.03633.x

[46] Kanehisa M, Goto S (2000) KEGG: Kyoto encyclopedia of genes and genomes. Nucleic Acids Research 28: 27–30. 10592173 10.1093/nar/28.1.27 PMC102409

[47] Cooley AM, Modliszewski JL, Rommel ML, Willis JH (2011) Gene duplication in *Mimulus* underlies parallel floral evolution via independent *trans*-regulatory changes. Current Biology 21: 700–704. 21474312 10.1016/j.cub.2011.03.028

[48] Streisfeld MA, Rausher MD (2009) Altered *trans*-regulatory control of gene expression in multiple anthocyanin genes contributes to adaptive flower color evolution in *Mimulus aurantiacus* . Molecular Biology and Evolution 26: 433–444. 19029190 10.1093/molbev/msn268

[49] Streisfeld MA, Rausher MD (2011) Population genetics, pleiotropy, and the preferential fixation of mutations during adaptive evolution. Evolution 65: 629–642. 21054357 10.1111/j.1558-5646.2010.01165.x

[50] Dubos C, Stracke R, Grotewold E, Weisshaar B, Martin C, et al (2010) MYB transcription factors in *Arabidopsis* . Trends in Plant Science 15: 573–581. 20674465 10.1016/j.tplants.2010.06.005

[51] Stracke R, Ishihara H, Barsch GHA, Mehrtens F, Niehaus K, et al (2007) Differential regulation of closely related R2R3-MYB transcription factors controls flavonol accumulation in different parts of the *Arabidopsis thaliana* seedling. Plant Journal 50: 660–677. 17419845 10.1111/j.1365-313X.2007.03078.x PMC1976380

[52] Preston J, Wheeler J, Heazlewood J, Li SF, Parish RW (2004) AtMYB32 is required for normal pollen development in *Arabidopsis thaliana* . Plant Journal 40: 979–995. 15584962 10.1111/j.1365-313X.2004.02280.x

[53] Williams EJB, Bowles DJ (2004) Coexpression of neighboring genes in the genome of *Arabidopsis thaliana* . Genome Research 14: 1060–1067. 15173112 10.1101/gr.2131104 PMC419784

[54] Chen W-H, de Meaux J, Lercher MJ (2010) Co-expression of neighbouring genes in *Arabidopsis*: separating chromatin effects from direct interactions. BMC Genomics 11: 178. 20233415 10.1186/1471-2164-11-178 PMC2851598

[55] Khaitovich P, Hellmann I, Enard W, Nowick K, Leinweber M, et al (2005) Parallel patterns of evolution in the genomes and transcriptomes of humans and chimpanzees. Science 309: 1850–1854. 16141373 10.1126/science.1108296

[56] Jeukens J, Renaut S, St-Cyr J, Nolte AW, Bernatchez L (2010) The transcriptomics of sympatric dwarf and normal lake whitefish (*Coregonus clupeaformis* spp., Salmonidae) divergence as revealed by next-generation sequencing. Molecular Ecology 19: 5389–5403. 21087448 10.1111/j.1365-294X.2010.04934.x

[57] Kohn MH, Shapiro J, Wu C-I (2008) Decoupled differentiation of gene expression and coding sequence among *Drosophila* populations. Genes and Genetic Systems 83: 265–273. 18670138 10.1266/ggs.83.265

[58] Abbott RJ, Brochmann C (2003) History and evolution of the arctic flora: In the footsteps of Eric Hulten. Molecular Ecology 12: 299–313. 12535083 10.1046/j.1365-294x.2003.01731.x

[59] Warwick SI, Al-Shehbaz IA (2006) Brassicaceae: Chromosome number index and database on CD-Rom. Plant Systematics and Evolution 259: 237–248.

[60] Bennett MD, Leitch IJ, Price HJ, Johnston JS (2003) Comparisons with *Caenorhabditis* (100 Mb) and *Drosophila* (175 Mb) using flow cytometry show genome size in *Arabidopsis* to be 157 Mb and thus 25% larger than the *Arabidopsis* Genome Initiative estimate of 125 Mb. Annals of Botany 91: 547. 12646499 10.1093/aob/mcg057 PMC4242247

