# Supplementary material for: Transcriptome Analysis of a Petal Anthocyanin Polymorphism in the Arctic Mustard, Parrya nudicaulis
Source: PLoS One. 2014 Jul 17;9(7):e101338. doi: 10.1371/journal.pone.0101338 (PMC4102464; doi:10.1371/journal.pone.0101338)
Supplement: File S1 — Supporting tables. Table S1, Fate of reads generated during the transcriptome assembly and expression analysis. Table S2, Genes with consistently higher expression in purple compared to white petal samples. Table S3, Genes with consistently higher expression in white compared to purple petal samples. (DOCX) [file pone.0101338.s001.docx]

**Supporting Information**

PONE-D-11-21875

“Transcriptome Analysis of a Petal Anthocyanin Polymorphism in the Arctic Mustard, *Parrya nudicaulis*”

Table S1. Fate of reads generated during the transcriptome assembly and expression analysis.

| Population | Color | Reads Generated | Reads Filtered Out^1^ | Reads Unaligned | Total Reads Aligned | Reads Multiply Aligned | Reads Uniquely Aligned^2^ |
| --- | --- | --- | --- | --- | --- | --- | --- |
| Savage River | Purple | 16,115,904 | 7,639,682 | 682,079 | 7,794,143 | 838,958 | 6,955,185 |
| Savage River | White | 15,292,542 | 7,404,300 | 833,217 | 7,055,025 | 762,493 | 6,292,532 |
| Twelve Mile | Purple | 26,204,412 | 13,014,324 | 2,067,396 | 11,122,692 | 1,247,278 | 9,875,414 |
| Twelve Mile | White | 28,734,954 | 13,475,305 | 3,960,608 | 11,299,041 | 1,261,580 | 10,037,461 |
| Totals |  | 86,347,812 | 41,533,611 | 7,543,300 | 37,270,901 | 4,110,309 | 33,160,592 |

^1^ Reads with greater than two mismatches were filtered out.

^2^ Only uniquely aligned reads were used in the expression analysis.

Table S2. Genes with consistently higher expression in purple compared to white petal samples.

|  |  | Savage River (RPKM^1^) | | 12 Mile Summit (RPKM) | |  |  |  |
| --- | --- | --- | --- | --- | --- | --- | --- | --- |
| TAIR Locus | Description | Purple | White | Purple | White | Mean RD^2^ | Consistency^3^ | P/W ratio |
| AT5G13930.1 | chalcone synthase/TT4 (transparent testa 4) | 4285.2 | 675.3 | 2334.1 | 302.4 | 0.86 | -0.03 | 7.03 |
| AT5G45950.1† | GDSL-motif lipase/ hydrolase family protein | 121.3 | 33.6 | 70.9 | 35.7 | 0.61 | 0.23 | 2.80 |
| AT4G19120.1 | ERD3 (early-responsive to dehydration 3) | 61.1 | 20.5 | 193.0 | 96.2 | 0.58 | 0.16 | 2.49 |
| AT1G06360.1 | fatty acid desaturase family protein | 204.6 | 112.8 | 23.4 | 7.2 | 0.57 | -0.24 | 2.53 |
| AT5G51750.1 | ATSBT1.3 (*Arabidopsis thaliana* subtilase 1.3) | 137.2 | 52.8 | 2.0 | 1.0 | 0.56 | 0.10 | 2.33 |
| AT1G29720.1 | protein kinase family protein | 195.7 | 63.5 | 174.7 | 99.5 | 0.55 | 0.25 | 2.42 |
| AT4G12270.1 | copper amine oxidase family protein | 13.4 | 6.8 | 152.6 | 65.3 | 0.53 | -0.08 | 2.15 |
| AT3G06550.3 | O-acetyltransferase family protein | 18.1 | 9.1 | 327.7 | 142.9 | 0.53 | -0.07 | 2.14 |

†Gene chosen for quantitative real-time PCR (qRT-PCR) validation

^1^ RPKM = Reads per kilobase exon per million uniquely mapped reads

^2^ Relative difference (RD) = (Purple RPKM – White RPKM)/max(Purple RPKM,White RPKM)

^3^ Consistency = RD_SavageRiver_ - RD_12MileSummit_

Table S3. Genes with consistently higher expression in white compared to purple petal samples.

|  |  | Savage River (RPKM^1^) | | 12 Mile Summit (RPKM) | |  |  |  |
| --- | --- | --- | --- | --- | --- | --- | --- | --- |
| TAIR Locus | Description | Purple | White | Purple | White | Mean RD^2^ | Consistency^3^ | W/P ratio |
| AT2G31141.1† | 7SL RNA gene | 12.2 | 255.3 | 5.6 | 20.6 | -0.84 | -0.22 | 12.29 |
| AT1G10020.1† | unknown protein | 33.2 | 118.9 | 17.6 | 44.2 | -0.66 | -0.12 | 3.04 |
| AT3G01950.1 | unknown protein | 112.7 | 255.7 | 7.6 | 25.9 | -0.63 | 0.15 | 2.85 |
| AT5G55930.1 | OPT1 (oligopeptide transporter 1) | 49.4 | 177.7 | 179.1 | 353.4 | -0.61 | -0.23 | 2.79 |
| AT5G62560.1 | armadillo/beta-catenin repeat family protein /U-box domain | 31.9 | 113.0 | 21.1 | 40.2 | -0.60 | -0.24 | 2.73 |
| AT4G13850.3 | GR-RBP2 (glycine-rich RNA-binding protein 2) | 335.5 | 1061.5 | 89.1 | 184.4 | -0.60 | -0.17 | 2.62 |
| AT3G13224.1 | RNA recognition motif (RRM)-containing protein | 49.2 | 168.0 | 19.9 | 38.1 | -0.59 | -0.23 | 2.66 |
| AT5G48540.1 | 33 kDa secretory protein-related | 33.7 | 68.2 | 27.2 | 84.0 | -0.59 | 0.17 | 2.56 |
| AT1G76080.1† | CDSP32 (cp drought-induced 32 kD stress-induced protein) | 40.2 | 112.2 | 59.3 | 126.2 | -0.59 | -0.11 | 2.46 |
| AT1G48760.2 | delta-ADR (delta-adaptin) | 54.8 | 161.8 | 43.9 | 88.7 | -0.58 | -0.16 | 2.49 |
| AT2G23810.1 | TET8 (tetraspanin8) | 69.0 | 144.6 | 27.2 | 75.3 | -0.58 | 0.12 | 2.43 |
| AT3G30775.1† | ERD5 (early responsive to dehydration 5) | 55.5 | 98.7 | 28.5 | 89.4 | -0.56 | 0.24 | 2.46 |
| AT2G44581.1 | protein binding / zinc ion binding | 23.7 | 68.5 | 61.2 | 114.2 | -0.56 | -0.19 | 2.38 |
| AT2G17230.1† | EXL5 (exordium like 5) | 547.5 | 1266.9 | 253.3 | 568.9 | -0.56 | -0.01 | 2.28 |
| AT4G20260.1 | DREPP plasma membrane polypeptide family protein | 19.4 | 45.8 | 55.6 | 121.5 | -0.56 | -0.03 | 2.28 |
| AT2G21180.1 | unknown protein | 36.7 | 65.9 | 43.5 | 123.3 | -0.55 | 0.20 | 2.32 |
| AT3G13310.1 | DNAJ heat shock N-terminal domain-containing protein | 32.8 | 80.5 | 49.4 | 102.2 | -0.55 | -0.08 | 2.26 |
| AT3G19680.1 | unknown protein | 82.1 | 211.5 | 71.5 | 137.2 | -0.55 | -0.13 | 2.25 |
| AT1G61760.1 | harpin-induced protein-related / HIN1-related | 67.3 | 163.9 | 7.5 | 14.6 | -0.54 | -0.10 | 2.19 |
| AT5G35735.1 | auxin-responsive family protein | 53.6 | 97.1 | 14.7 | 37.4 | -0.53 | 0.16 | 2.18 |
| AT5G15410.2† | DND1 (defense no death 1) | 93.5 | 198.9 | 162.4 | 352.4 | -0.53 | 0.01 | 2.15 |
| AT3G47340.1 | ASN1 (glutamine-dependent asparagine synthase 1) | 82.9 | 164.4 | 14.5 | 32.9 | -0.53 | 0.07 | 2.13 |
| AT1G02170.1 | AMC1 (metacaspase 1) | 41.1 | 111.5 | 24.0 | 40.7 | -0.52 | -0.22 | 2.20 |
| AT2G26740.1 | ATSEH (*Arabidopsis thaliana* soluble epoxide hydrolase) | 31.6 | 64.1 | 63.6 | 132.1 | -0.51 | 0.01 | 2.05 |
| AT5G16570.1 | GLN1;4; glutamate-ammonia ligase | 52.6 | 89.2 | 23.3 | 57.8 | -0.50 | 0.19 | 2.09 |

†Genes chosen for quantitative real-time PCR (qRT-PCR) validation

^1^ RPKM = Reads per kilobase exon per million uniquely mapped reads

^2^ Relative difference (RD) = (Purple RPKM – White RPKM)/max(Purple RPKM,White RPKM)

^3^ Consistency = RD_SavageRiver_ - RD_12MileSummit_
